# The Intersection of Diabetes and Cardiovascular Disease—A Focus on New Therapies

**DOI:** 10.3389/fcvm.2018.00160

**Published:** 2018-11-13

**Authors:** Devinder S. Dhindsa, Pratik B. Sandesara, Michael D. Shapiro

**Affiliations:** ^1^Division of Cardiology, Department of Medicine, Emory University School of Medicine, Atlanta, GA, United States; ^2^Center for Preventive Cardiology, Knight Cardiovascular Institute, Oregon Health & Science University, Portland, OR, United States

**Keywords:** diabetes, cardiovascular disease, cardiovascular outcomes trials, SGLT-2 inhibitors, GLP1-receptor agonists

## Abstract

Diabetes is a leading cause of cardiovascular disease and its associated morbidity. While the medical community has had access to numerous glucose lowering therapies over the last decades, it was not until recently that newer agents demonstrated improvement in cardiovascular outcomes. In particular, diabetes care and management of its attendant cardiovascular risk is now being revolutionized with the development and provision of the SGLT-2 inhibitors and GLP1-receptor agonists. Given the exciting data with these new classes of diabetes therapeutics, there is a clear need to improve education and utilization of these evidence-based medications across a wide spectrum of clinicians, including cardiologists. The aim of this review is to familiarize the cardiovascular specialist with the benefits and harms of the most commonly used oral anti- hyperglycemic medications, with an emphasis on SGLT-2 inhibitors and GLP-1 receptor agonists.

## Introduction

Eighty percent of individuals with Type 2 diabetes mellitus (DM) will ultimately succumb to death from a cardiovascular cause compared to 30% of the non-diabetic population (1). The adjusted relative risk (RR) for DM compared to non-DM patients is 1.7 for cardiovascular death, 1.8 for myocardial infarction (MI), and are 1.5 for stroke (2). DM confers a high lifetime risk (67% in men and 57% in women) for developing CVD (3). This association is further compounded by the fact that metabolic risk factors for atherosclerotic CVD (ASCVD) are commonly found in patients with diabetes (4).

Unfortunately, the role of glycemic control in prevention of ASCVD and macrovascular disease is complex and has not shown a clear benefit in preventing these outcomes. In the United Kingdom Prospective Diabetes Study (UKPDS) of 5,102 individuals with newly diagnosed DM, intensive glycemic control (treatment to A1C < 7%) did not improve macrovascular outcomes as compared to the standard control arm (A1C < 9%) (5). Additionally, meta-analyses of the Action to Control Cardiovascular Risk study (ACCORD), Action in Diabetes and Vascular Disease: Preterax and Diamicron Modified Release Controlled Evaluation trial (ADVANCE), and Veteran Affairs Diabetes Trial (VADT) demonstrated that intensive therapy, compared to conventional therapy, is associated with adverse events, namely hypoglycemia without a significant reduction in cardiovascular events despite a reduction in microvascular complications (6). These findings were likely mediated by an increase in hypoglycemic events in the intensive therapy groups.

Concerns regarding the cardiovascular safety of diabetes medications (e.g., rosiglitazone) prompted the U.S. Food and Drug Administration (FDA) to issue a mandate in 2008 that required dedicated large cardiovascular safety trials as part of all diabetes drug development programs. Specifically, the FDA guidance provided recommendations on how to demonstrate that a new antidiabetic therapy is not associated with an unacceptable increase in cardiovascular risk. After several years of trials of mainly dipeptidyl peptidase-4 inhibitors (DPP-IV) failing to show cardiovascular benefit, the mandate eventually lead to the discovery of the cardiovascular benefits of sodium-glucose cotransporter-2 (SGLT-2) inhibitors and glucagon-like peptide 1 (GLP-1) receptor agonists, particularly in those at highest risk for CVD. These medications demonstrate improvement in cardiovascular outcomes and mortality through mechanisms that likely have little to do with their glucose lowering effects. The aim of this review is to familiarize cardiovascular specialists with the medical management of DM with respect to cardiovascular benefit and risk, with an emphasis on these two new classes of medications.

## Pharmacologic therapy

The primary goal for glucose management in patients with type 2 diabetes has long been targeting a hemoglobin A1C of <7.0%. While achievement of optimal hemoglobin A1C levels has been associated with a reduction in microvascular complications, randomized controlled trials have failed to demonstrate a benefit in preventing macrovascular outcomes with this strategy (7, 8). Contemporary trials with new classes of anti-diabetic medications that demonstrate cardiovascular benefit despite only modest glucose lowering challenge this dogma and point to medication class (e.g., mechanism of action) being an important driver of outcomes. The most common classes of diabetes medications, their mechanism of actions, and adverse effects are summarized in Table 1 (9). What follows is a consideration of the various classes of diabetes medications that are associated with either favorable, neutral, or unfavorable cardiovascular outcomes.

**Table 1:** Common oral hypoglycemic medications.

	**Mechanism of action**	**Examples**	**Approximate A1C reduction (%)**	**Impact on CV events**	**Adverse effects**
Biguanides	Activates AMPK	Metformin	1–2	Reduction in MI, all-cause mortality	diarrhea, nausea, lactic acidosis
Sulfonylureas	Increase insulin secretion via ATP-sensitive K channel on beta cells	Glimepiride, Glipizide, Glyburide	1–2	No effect; risk of hypoglycemia	hypoglycemia, weight gain
DPP-IV inhibitors	Prevents degradation of GLP-1	Saxagliptin, Sitagliptin, Vildagliptin	0.5–0.8	Increased heart failure hospitalization for saxagliptin	Nausea (generally resolves)
Thiazolidinediones	Bind PPAR gamma, decrease insulin resistance and increase glucose utilization	Rosiglitazone, Pioglitazone	0.5–1.4	Increased risk of heart failure; pioglitazone may be associated with reduced MACE	Peripheral edema, HF, weight gain, fractures
SGLT-2 Inhibitors	Block glucose resorption in proximal renal tubule	Canagliflozin, Empagliflozin, Dapagliflozin, Ertugliflozin	0.5–0.8	Reduction in HF hospitalization, CV mortality	GU infections, increased lower extremity amputation with canagliflozin (0.6% v 0.3% in placebo)
GLP-1 Agonists	Activated glucagon-like-peptide 1 receptor, increasing insulin secretion, decreasing glucagon selection	Liraglutide, Semaglutide, Exenatide	0.4–0.9	Reduction in CV mortality, all-cause mortality, MI/stroke	GI side effects. Higher rates of retinopathy with semaglutide

*AMPK, 5' adenosine monophosphate-activated protein kinase; MI, myocardial infarction; CV, cardiovascular; MACE, major adverse cardiovascular event; ATP, adenosine triphosphate; K, potassium, DPP-IV, dipeptidyl peptidase 4; GLP-1, glucagon like peptide 1; PPAR, peroxisome proliferator-activated receptor; HF, heart failure; SGLT-2, sodium-glucose cotransporter 2; GU, genitourinary; GI, gastrointestinal*.

## Medications with *Favorable* CV outcomes

In the absence of contraindications, these drugs *should be considered* in eligible patients with cardiovascular disease and diabetes given the favorable cardiovascular outcome trial data associated with their use (10) (Table 2).

**Table 2:** Cardiovascular outcomes in select CVOTs.

**Trial**	**Drug**	**Drug class**	**Number of patients**	**Median duration (years)**	**Primary endpoint**	**CV death**	**MI**	**HF hospitalization**	**All-cause mortality**
EMPA-REG	Empagliflozin	SGLT2-i	7,020	3.1	3-point MACE HR 0.86 (0.74–0.99)	HR 0.62 (0.49–0.77)	HR 0.87 (0.70–1.09)	HR 0.65 (0.50–0.85)	HR 0.68 (0.57–0.82)
CANVAS	Canagliflozin	SGLT2-i	10,142	2.4	3-point MACE HR 0.86 (0.75–0.97)	HR 0.87 (0.72–1.06)	HR 0.89 (0.73–1.09)	HR 0.67 (0.52–0.87)	HR 0.87 (0.74–1.01)
LEADER	Liraglutide	GLP-1ra	9,340	3.8	3-point MACE HR 0.87 (0.78–0.97)	HR 0.78 (0.66–0.93)	HR 0.86 (0.73–1.00)	HR 0.87 (0.73–1.05)	HR 0.85 (0.74–0.97)
SUSTAIN-6	Semaglutide	GLP-1ra	3,297	2.1	3-point MACE HR 0.74 (0.58–0.95)	HR 0.98 (0.65–1.48)	HR 0.74 (0.51–1.08)	HR 1.11 (0.77–1.61)	HR 1.05 (0.74–1.50)
HARMONY	Albiglutide	GLP-1ra	9,463	1.5	3-point MACE HR 0.78 (0.68–0.90)	HR 0.93 (0.73–1.19)	HR 0.75 (0.61–0.90)	HR 0.85 (0.70–1.04) (composite of CV death and HF hospitalization)	HR 0.95 (0.79–1.16)

*MACE, major adverse cardiovascular event; 3-point MACE: CV death, non-fatal MI, non-fatal stroke; CV, cardiovascular; MI, myocardial infarction; HF, heart failure, HR, hazard ratio; GLP-1ra, glucagon like peptide 1 receptor agonist; SGLT2-i, sodium-glucose cotransporter 2 inhibitor*.

### Biguanides

Metformin is the preferred initial medication for the treatment of type 2 diabetes per current American Diabetes Association (ADA) recommendation (7). This medication exerts its glucose lowering effects by decreasing hepatic gluconeogenesis and increasing insulin sensitivity. In a substudy of the UKPDS that evaluated obese patients, a relative risk reduction of 39% in MI in individuals on metformin was seen as compared to those on insulin or sulfonylureas (8). Additionally, metformin was associated with a 24% reduction in all-cause death (95% CI: 0.65–0.89, *P* < 0.001) as compared to those not on the medication (11). This evidence supports metformin as first-line therapy for type 2 diabetes given its relative safety and beneficial effects on hemoglobin A1C, weight, and cardiovascular morbidity (12). The mechanism of cardiovascular benefit of these medications is derived mainly from murine models. In murine models of MI, the administration of metformin limits infarct size (13).

Experimentally, this has been found to be due to activation of adenosine monophosphate-activated protein kinase, increased formation of adenosine, and the prevention of opening of the mitochondrial permeability transition pore at reperfusion that contribute to this effect.

Additionally, metformin also attenuates post-infarction cardiac remodeling through mechanisms including the activation of adenosine monophosphate-activated protein kinase and endothelial nitric oxide synthase, and reduced collagen expression (14).

Contraindications for the clinician to be aware of are renal failure (estimated glomerular filtration rate >30 mL/min/1.73 m^2^) as well as decompensated heart failure given the risk of lactic acidosis (7). Additionally, it is standard of care to discontinue metformin during periods of renal impairment or inpatient heart failure treatment, as well as 24 h prior to and 48 h following contrast exposure (7).

### SGLT-2 inhibitors

The SGLT-2 inhibitors antagonize the sodium-glucose cotransporter 2, located in the proximal tubule of the kidneys. This cotransporter is responsible for 90% of the glucose reabsorption occurring in the kidney (15). Thus, inhibition of this cotransporter leads to glucosuria, which is the predominant anti-hyperglycemic mechanism for these medications.

**Figure 1:**
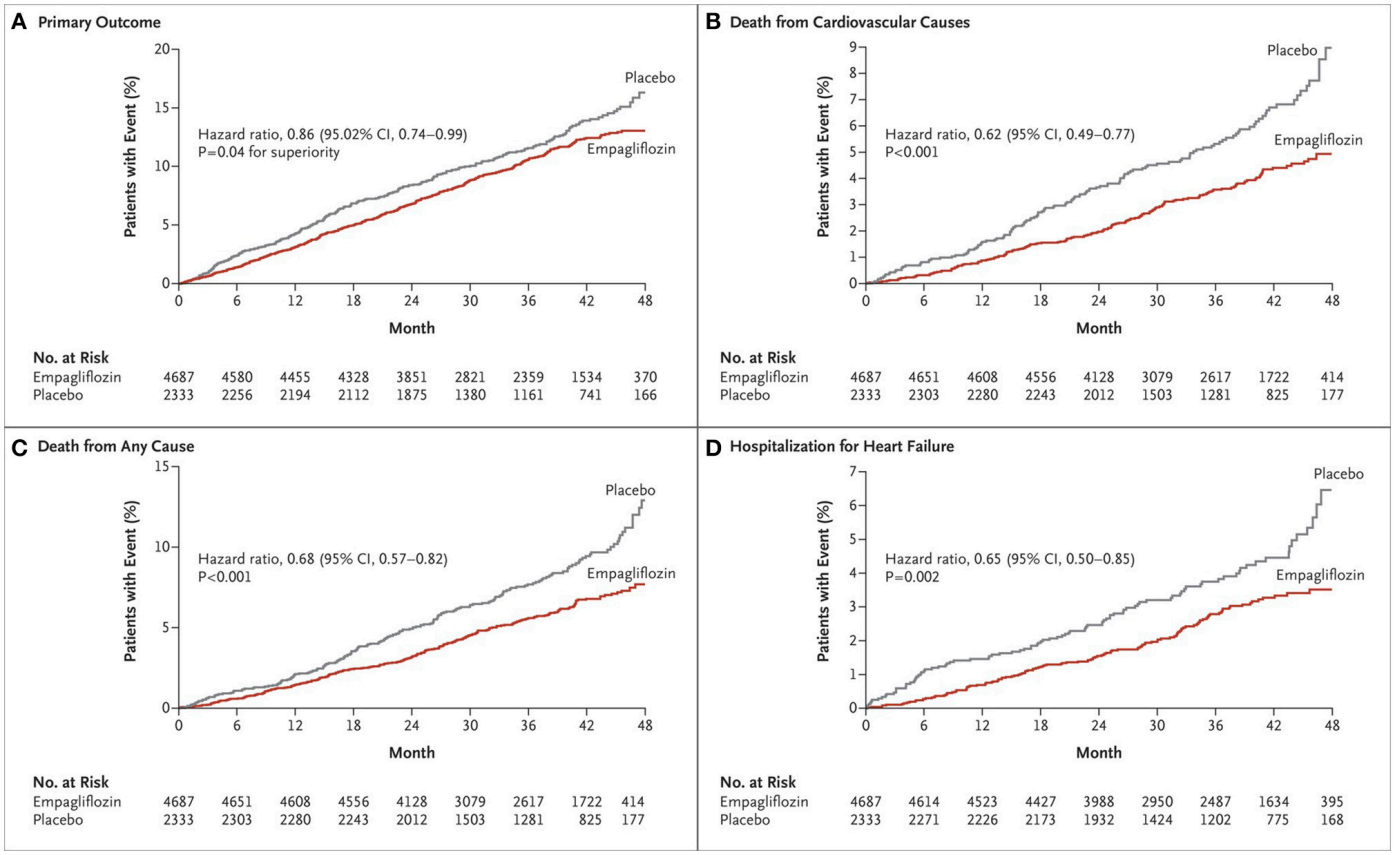
Cardiovascular outcomes and death from any cause in EMPA-REG OUTCOME (16). Cumulative incidence of the primary outcome (death from cardiovascular causes, nonfatal myocardial infarction, or nonfatal stroke) **(A)**; Cumulative incidence of death from cardiovascular causes **(B)**; Kaplan-Meier estimate for death from any cause **(C)**; Cumulative incidence of hospitalization for heart failure **(D)**. Copyright 2015. Massachusetts Medical Society. Reprinted with permission from Massachusetts Medical Society.

The first SGLT-2 inhibitor studied in a dedicated cardiovascular outcomes trial was empagliflozin in the Empagliflozin Cardiovascular Outcome Event Trial in Type 2 Diabetes Mellitus Patients- Removing Excess Glucose (EMPA-REG OUTCOME) (16). In EMPA-REG OUTCOME, 7,020 patients were followed over a median follow-up of 3.1 years. This study was a randomized, double-blind trial that compared empagliflozin with placebo in a population with diabetes and known CVD. The primary composite outcome of MI, stroke, and cardiovascular death was reduced by 14% (HR: 0.86 in empagliflozin group, 95% CI: 0.74–0.99, *p* = 0.04) with a reduction in cardiovascular death of 38% (HR: 0.62, 95% CI: 0.49–0.77, *P* < 0.001) (Figure 1). Additionally, subjects in the empagliflozin arm demonstrated a 35% reduction in heart failure hospitalization as compared to placebo (HR: 0.65, 95% CI: 0.50–0.85, *p* = 0.002). Because of the strength of this data, the FDA has included reduction of risk of cardiovascular death in adults with type 2 diabetes and CVD as an indication for empagliflozin (17).

Similar findings were replicated in the Canagliflozin Cardiovascular Assessment Study (CANVAS) which investigated another SGLT-2 inhibitor, canagliflozin vs. placebo (18). The trial enrolled 10,142 patients with type 2 DM and high cardiovascular risk with a median follow-up of 3.6 years. Like EMPA-REG OUTCOME, CANVAS demonstrated a significant reduction in the composite outcome of cardiovascular death, MI, or stroke in the canagliflozin group as compared to placebo (HR: 0.86, 95% CI: 0.75–0.97, *P* < 0.001 for non-inferiority, *P* = 0.02 for superiority). However, there was not a statistically significant difference in the individual components of cardiovascular death (HR: 0.87, 95% CI: 0.72–1.06), MI (HR: 0.85, 95% CI: 0.69–1.05), or stroke (HR: 0.90, 95% CI: 0.71–1.15). All-cause mortality was also not significantly reduced (HR: 0.87, 95% CI: 0.74–1.01). Heart failure hospitalizations were significantly reduced in the canagliflozin arm by 33% (HR: 0.67, 95% CI: 0.52–0.87). Of note, there was also an increased risk of amputation with canagliflozin (6.3 vs. 3.4 events per 1,000 patient-years; HR: 1.97, 95% CI: 1.41–2.75).

Interestingly, the difference in mortality and cardiovascular endpoints occurred relatively early (within the first few weeks) in these trials. The possible explanations for these observations include osmotic diuresis leading to improved cardiac hemodynamics by reduction in left ventricular afterload, lowering of body weight due to calorie and fluid losses, and lowering of blood pressure (19, 20). Heart failure hospitalizations were reduced in the SGLT-2 inhibitor arm of the CANVAS and EMPA-REG OUTCOME trials, likely due to this osmotic diuretic effect. Another proposed mechanism of cardiovascular benefit may be a shift in fuel energetics to the myocardial cells via increased ketosis (with a rare but serious adverse event of non-hyperglycemic ketoacidosis), which is a preferred substrate for cardiac tissue, as opposed to free fatty acids (21). This improvement in metabolic efficiency is theorized to translate to CVD benefit. Another important finding seen in these studies was the improvement in “hard” renal outcomes. In both canagliflozin and empagliflozin, there was an improvement, as compared to placebo, in the composite outcome of >40% reduction in GFR, need for renal replacement therapy, or death resulting from renal disease (HR: 0.60, 95% CI: 0.47–0.77 for canagliflozin, HR: 0.61, 95% CI: 0.53–0.70 for empagliflozin) (18, 22). These medications were also noted to reduce the progression ofalbuminuria in canagliflozin in CANVAS (HR: 0.73, 95% CI: 0.67–0.79), though the rate of incident albuminuria was not different between placebo and empagliflozin in EMPA- REG. The proposed mechanism for improved renal outcomes is unclear, though it is suspected to be secondary to decreased plasma volume with concomitant reduction in hyperfiltration and intraglomerular pressure (23).

Importantly, a multinational, observational study in 306,156 adults with type 2 DM and only 13% prevalence of established CVD was undertaken in the Comparative Effectiveness of Cardiovascular Outcomes in New Users of SGLT-2 Inhibitors (CVD-REAL) Study (24). This population was in contrast to the study populations of EMPA-REG OUTCOME and CANVAS, who had established CVD or were otherwise at high CV risk. The main medications used in the study were canagliflozin (53%), dapagliflozin (42%), and empagliflozin (5%). SGLT-2 inhibitors were associated with a lower risk of death in individuals with and without CVD (HR: 0.56, 95% CI: 0.44–0.70; HR: 0.56, 95% CI: 0.50–0.63) as well as a reduction in heart failure hospitalizations (HR: 0.72, 95% CI: 0.63–0.82; HR: 0.61, 95% CI: 0.48–0.78). The importance of the mortality benefit noted even in patients without established CVD at baseline cannot be overstated and is a finding that has not been observed with any other class of anti-diabetic medications. Additionally, there was no significant heterogeneity between the effects among the SGLT-2 inhibitor used, indicating the observed benefits are likely a class effect. The Multicenter Trial to Evaluate the Effect of Dapagliflozin on the Incidence of Cardiovascular Events (DECLARE-TIMI 58) is ongoing and is investigating the cardiovascular outcomes of dapagliflozin in patients with established CVD or risk factors for CVD.

There are several important side effects for patients to be aware of prior to initiating treatment with an SGLT-2 inhibitor. An increased rate of genital mycotic infections, volume depletion and dehydration, and increased urinary tract infections are likely secondary to the mechanism of action of this class (glucosuria leading to osmotic diuresis) (25). Additionally, there is a rare but serious, side effect of euglycemic diabetic ketoacidosis as discussed previously. Briefly, this complication is, in part, a result of persistent glycosuria triggering a sequence of metabolic changes that leads to decreased insulin production and increased glucagon secretion, stimulating enhanced ketogenesis (26). This potential adverse event requires vigilance by the clinician as the lack of significant hyperglycemia can lead to delayed or missed diagnosis of diabetic ketoacidosis. Canagliflozin was also associated with a small but statistically significant increase in the risk of bone fractures and amputation. The exact mechanism of the higher rates of amputation with canagliflozin is unknown, though a proposed mechanism is volume depletion leading to circulatory failure in distal peripheral arterial beds (27). This has led to an FDA warning regarding avoiding canagliflozin in individuals at risk for amputation, namely those with a history of prior amputation, peripheral vascular disease, neuropathy, and diabetic foot ulcers (28). These medications are contraindicated in signficant renal disease (GFR < 30 ml/min) and should be used with caution in the elderly and frail given the risk of volume depletion (29).

### GLP-1 agonists

The GLP-1 receptor agonists, liraglutide and semaglutide, have also demonstrated cardiovascular benefits in individuals with diabetes and high-risk for CVD. GLP-1 receptor agonists exert their anti-hyperglycemic effect by potentiating insulin secretion, decreasing postprandial glucagon, delaying gastric emptying, and promoting weight loss (30). GLP-1, along with glucose-dependent insulinoptropic polypeptide (GIP), is responsible for the incretin effect, which is the augmentation of insulin secretion post-prandially after the oral ingestion of glucose (31).

**Figure 2:**
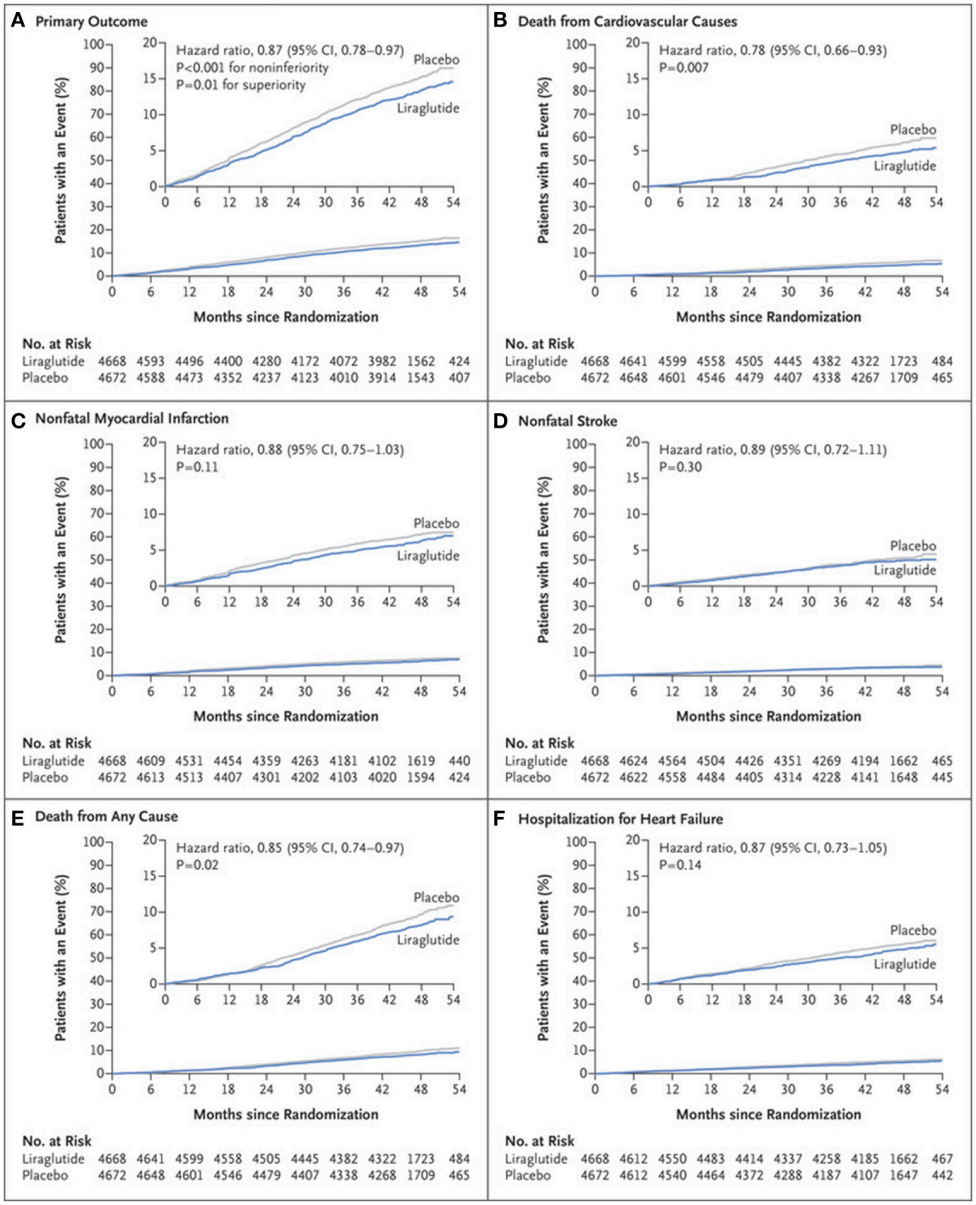
Primary and exploratory outcomes in LEADER trial (32). Cumulative incidence of primary composite outcome **(A)**; Cumulative incidence of death from cardiovascular causes **(B)**; Cumulative incidence of nonfatal myocardial infarction **(C)**; Cumulative incidence of nonfatal stroke **(D)**; Cumulative incidence of death from any cause **(E)**; Cumulative incidence of hospitalization for heart failure **(F)**. Copyright 2016. Massachusetts Medical Society. Reprinted with permission from Massachusetts Medical Society.

In the Liraglutide Effect and Action in Diabetes: Evaluation of Cardiovascular Outcomes Results-A Long Term Evaluation (LEADER) trial, 9,340 patients with type 2 DM and high-risk for CVD or with known CVD were followed over 3.8 years (32). The primary outcome of CV death, non-fatal MI, or non-fatal stroke occurred in 13.0% of patients in the group randomized to liraglutide as compared to 14.9% in the control arm (HR: 0.87, 95% CI: 0.78–0.97, *P* < 0.001 for non-inferiority, *P* = 0.01 for superiority) (Figure 2). Cardiovascular death was significantly reduced in the liraglutide group (4.7 vs. 6.0%, HR: 0.78, 95%CI: 0.66–0.93, *P* = 0.007). Though the other components of the primary endpoint were numerically in favor of liraglutide, they were not found to be statistically significant (non-fatal MI: HR: 0.88, 95% CI: 0.75–1.03, *p* = 0.11; non-fatal stroke: HR: 0.89, 95% CI: 0.72–1.11, *p* = 0.30). Additionally, all-cause mortality was reduced in the liraglutide arm (HR: 0.85, CI: 0.74–0.97, *p* = 0.02). The liraglutide arm also had an improvement in A1C (−0.40%, 95% CI: −0.45 to −0.34%), weight (−2.3 kg, 95% CI: −2.0 to −2.5 kg), and systolic blood pressure (−1.2 mmHg, 95% CI: −0.5 to −1.9 mmHg). Given the improvement in the primary endpoint seen with liraglutide, the FDA approved its use for reducing the risk of major adverse cardiovascular events (MACE) in individuals with type 2 DM and known CVD in 2017 (33).

In the Semaglutide in Subjects with Type 2 Diabetes (SUSTAIN-6) study, 3,297 patients with CVD or at high risk were randomized to semaglutide, at 0.5 or 1.0 mg, or placebo for 104 weeks (34). A 26% reduction in the primary endpoint of CV death, non-fatal MI, and non-fatal stroke was observed in subjects randomized to semaglutide (HR: 0.74, 95% CI: 0.58–0.95, *P* < 0.001). However, in contrast to LEADER, CV mortality was not significantly reduced (HR: 0.98, 95% CI: 0.65–1.48, *p* = 0.92), though non-fatal stroke was improved (HR: 0.61, 95% CI: 0.38–0.99, *p* = 0.04) along with a non-significant trend to lower rates of MI (HR: 0.74, 95% CI: 0.51–1.08, *p* = 0.12). Similar to LEADER, as compared to placebo there was an improvement in glycemic control (A1C −0.7 and −1.0% in those receiving 0.5 and 1.0 mg, respectively; *p* < 0.001 for both groups), body weight (−2.9 kg in those receiving 0.5 mg semaglutide, −4.3 kg in those receiving 1.0 mg; *p* < 0.001 for both groups), and systolic blood pressure (−1.3 mmHg in the 0.5 mg group, −2.6 mmHg in the 1.0 mg group; *p* = 0.10 and < 0.001, respectively). Importantly, more patients in the semaglutide group stopped treatment due to adverse events (12.9 vs. 6.7% in placebo), which were namely gastrointestinal complaints. Additionally, there appeared to be a higher rate of retinopathy complications in the semaglutide arm (HR: 1.76, 95%CI: 1.11–2.78, *p* = 0.02). The mechanism of this is thought due to worsening of pre-existing diabetic retinopathy associated with rapid and large improvements of glycemic control, a phenomenon noted with insulin as well and not thought to be a deleterious effect of semaglutide (35).

Additionally, there is recent evidence of another GLP1 receptor agonists, albiglutide, showing CV benefit. In the Albiglutide and Cardiovascular Outcomes in Patients with Type 2 Diabetes and Cardiovascular Disease (HARMONY) trial, 10,793 patients were followed for a median of 1.8 years (36). This trial demonstrated a reduction in the composite primary outcomes of CV death, MI, or stroke (HR: 0.78, 95% CI: 0.68–0.90, *p* < 0.0001 for non-inferiority; *p* = 0.0006 for superiority). In the individual components, there was no difference in CV death or stroke, though there was a significant reduction in MI (HR: 0.75, 95% CI: 0.61–0.90, *p* = 0.003). There was no difference in heart failure hospitalization or all-cause mortality between albiglutide and placebo.

There are several important considerations within these trials. The reduction in the primary end point was driven by significantly lower CV mortality in LEADER. While there was numerical reduction in MI and stroke rates, this did not reach statistical significance. SUSTAIN- 6 also showed composite CV event reduction with semaglutide, which was driven by a significant reduction in stroke (1.6 vs. 2.7% in placebo; *P* = 0.04) rather than CV mortality. HARMONY did not show any mortality benefit to albiglutide as compared to placebo, though did show a statistically significant reduction in MI. Neither liraglutide, albiglutide, or semaglutide had a significant effect on HF admissions, suggesting a different mechanism of action than SGLT2 inhibitors. Though not completely understood, it has been suspected that these drugs may have more of an anti-atherothrombotic effect given the end points improved (MI, stroke) as well as the timing of the benefit seen later in the trials (months to years) in contrast to when the benefits were seen with SGLT-2 inhibitors (weeks). However, given the magnitude of the CV mortality improvement seen in LEADER, if an anti-atherogenic mechanism was primarily responsible, a more convincing reduction in MI or stroke would be expected (neither were significantly reduced in LEADER). Other potential effects like blood pressure lowering, weight reduction and avoidance of hypoglycemia may be contributory to improved CV outcomes (37).

There are currently five GLP-1 agonists that are available for clinical use. These agents produce significant improvement in glycemic control in association with modest weight loss. Thus far, only liraglutide 1.2 to 1.8 mg SQ daily has been approved by the FDA with an indication for reducing the risk of cardiovascular events in individuals with T2DM and CVD. Lixisenatide and Exenatide are two other GLP-1 agonists that have also been scrutinized within the context of CVOTs. However, neither of these drugs demonstrated superiority over placebo to reduce the composite endpoint of stroke, MI, and cardiovascular death that was seen in LEADER and SUSTAIN-6 (38, 39).

The most common side effects with GLP-1 agonists are gastrointestinal in nature, namely diarrhea and vomiting. These side effects occur early, but tend to be transient (40). Also given that this class of medications is renally excreted, a GFR < 30 ml/min is a contraindication to this therapy.

## Medications with no effect on CV outcomes

Insulin and sulfonylureas are two medications that have not demonstrated CV benefit. They should be considered as second- or third-line agents, after having prioritized the medications that have demonstrated improvement in CV outcomes (i.e., metformin, SGLT-2 inhibitors, GLP-1 receptor agonists).

### Insulin

Subcutaneous insulin therapy should be considered in patients with: (1) renal or hepatic impairment that precludes the safe use of an oral hypoglycemic, (2) individuals failing to reach their glycemic target on oral hypoglycemics alone, (9) and/or (3) if initiation of treatment is occurring in the inpatient setting (41). Insulin therapy is eventually required by a significant proportion of individuals with type 2 DM given the progressive decrease in insulin production associated with long-standing type 2 DM (42). Generally glucose control is improved by insulin therapy in patients who do not reach their glycemic target on alternative regimens (9). Improved glycemic control has demonstrated improvement in microvascular disease (i.e., retinopathy, nephropathy, neuropathy) (43). Unfortunately, the Outcome Reduction with an Insulin Glargine Intervention (ORIGIN) trial did not show a reduction in macrovascular outcomes when a strategy of early implementation of subcutaneous insulin-based therapies were employed (43). Additionally, the risk of hypoglycemia is a major adverse effect of insulin therapy that can lead to worsened cardiovascular outcomes and death (44).

### Sulfonylureas

Sulfonylureas are the oldest class of oral glucose-lowering agents. These medications exert their anti-hyperglycemic effect by increasing endogenous insulin secretion via the ATP- sensitive K channel on beta cells (9). Although the first generation of sulfonylureas exhibited an increase in CV and all-cause mortality, this observation has not been recapitulated with the second and third generation sulfonylureas. The major concern with this class relates to its main side effects of hypoglycemia, an independent contributor to CV death, and weight gain (45). For these reasons, sulfonylureas should be considered second- or third-line agents for the treatment of DM in individuals with cardiovascular disease. In those without CVD, sulfonylureas are considered a second line agent according to the joint American Diabetes Association and European Association for the Study of Diabetes Consensus algorithm (9).

## Medications that may have an *Unfavorable* effect on CV outcomes

### Thiazolidinediones

Several oral hypoglycemics should be avoided in those with cardiovascular disease, namely the thiazolidinediones, rosiglitazone, and pioglitazone. Peripheral edema is a noted side effect of this drug class, mediated by increased sodium reabsorption by the renal peroxisome proliferator-activated receptor γ-dependent pathway in the collecting tubules leading to increased plasma volume and subsequent fluid overload (46). Both rosiglitazone and pioglitazone have been associated with an increased risk of heart failure (47, 48). Additionally, rosiglitazone had previously been associated with an increased risk of MI and cardiovascular mortality when evaluated in a 2007 meta-analysis of 42 trials, though this has been a source of contention as other analyses do not suggest safety issues with this agent (49). Nonetheless, the question of increased CV events and mortality lead to the 2008 FDA mandate requiring dedicated safety trials as part of the development programs of new diabetes drugs, as discussed previously. Another thiazolidinedione, pioglitazone, did not show the initial concerning signals that rosiglitazone was thought to show (50). In fact, recent evidence has indicated that pioglitazone may be associated with a reduced risk of MACE, though the risks of heart failure and bone fracture were increased (49). As such, thiazolidinediones should be avoided in those with or at risk of heart failure given the concerns related to sodium and water retention.

### Dipeptidyl peptidase-4 inhibitors

The safety of DPP-4 inhibitors was evaluated in several CVOTs following the FDA mandate in 2008. In the Saxagliptin Assessment of Vascular Outcomes Recorded in Patients with Diabetes Mellitus-Thrombolysis in Myocardial Infarction 53 (SAVOR-TIMI 53), the Effect of Sitagliptin on Cardiovascular Outcomes in Type 2 Diabetes (TECOS), andthe Examination of Cardiovascular Outcomes with Alogliptin vs. Standard of Care in Patients with Type 2 Diabetes Mellitus and Acute Coronary Syndromes (EXAMINE) trials, saxagliptin, sitagliptin, and alogliptin exhibited similar rates of CVD events as compared to placebo (51–53). However, hospitalization for heart failure in individuals treated with saxagliptin was significantly higher as compared to placebo (3.5 vs. 2.8%; hazard ratio, 1.27; 95% CI: 1.07–1.51; *P* = 0.007).

Alogliptin and sitagliptin, however, showed a neutral effect on heart failure outcomes. There has not been any cardiovascular benefit observed with this class. In fact, the potential harm related to an increase in heart failure hospitalizations has led to a recommendation away from using these drugs in individuals at risk for CVD, though this is a topic of debate and remains to be further studied.

## Future directions

The above data, in conjunction with the lack of cardiovascular or mortality benefit seen with glycemic control alone, should alter the established paradigm of glycemic control as the pillar of DM treatment. The slavish reliance of targeting a hemoglobin A1C to <7 has not been fruitful for managing macrovascular risk (54). While managing dysglycemia is important for mitigating microvascular risk, no data demonstrate meaningful improvements in cardiovascular outcomes with aggressive glucose control (4–6, 8). Two new classes of drugs, the SGLT-2 inhibitors and GLP1-receptor agonists, show great promise in transforming the treatment of diabetes by independently improving cardiovascular outcomes, above and beyond what can be achieved with standard of care management. Nonetheless, a great deal remains to be discovered in terms of the mechanisms by which these drugs elicit their positive effects.

While the new diabetes drugs have ushered in an exciting new era of managing cardiovascular risk, additional CVOTs are needed. The landmark studies with the SGLT-2 inhibitors and GLP-1 receptor agonists enrolled only high-risk patients with DM and CVD. The utility and safety of these medications in the general population is an important question that remains to be answered. The impact of these medications is likely to be different when used in in lower risk patients. Additionally, the evaluation of their safety is an important step in determining the risk and benefit calculations clinicians rely on while individualizing treatment. Trials such as DECLARE-TIMI58, VERTIS CV, and SCORED investigating dapagliflozin, ertugliflozin, and sotagliflozin, respectively, are currently under way for the investigation of novel SGLT2-inhibitors. Additionally, PIONEER 6 and REWIND trials investigating semaglutide and albiglutide, respectively, are ongoing as well (55). These medications have shown such a great effect that trials are currently underway to investigate their effects in heart failure populations without overt DM, as in the EMPEROR trial with empagliflozin and the DAPA-HF trial with dapagliflozin. We are at the dawn of a new era in the management of diabetes and cardiovascular risk, and the future is bright!

## Author contributions

All authors listed have made a substantial, direct and intellectual contribution to the work, and approved it for publication.

### Conflict of interest statement

MS reports advisory activities with the following companies: Regeneron, Novartis, Esperion. The remaining authors declare that the research was conducted in the absence of any commercial or financial relationships that could be construed as a potential conflict of interest.
